# Image-Based In Vitro Screening Reveals the Trypanostatic Activity of Hydroxymethylnitrofurazone against *Trypanosoma cruzi*

**DOI:** 10.3390/ijms22136930

**Published:** 2021-06-28

**Authors:** Cauê Benito Scarim, Francisco Olmo, Elizabeth Igne Ferreira, Chung Man Chin, John M. Kelly, Amanda Fortes Francisco

**Affiliations:** 1 School of Pharmaceutical Sciences, Sao Paulo State University (UNESP), Araraquara, Sao Paulo 14800-903, Brazil; cauebenitos@gmail.com (C.B.S.); chungmanchin@gmail.com (C.M.C.); 2 Department of Infection Biology, Faculty of Infectious and Tropical Diseases, London School of Hygiene and Tropical Medicine, Keppel Street, London WC1E 7HT, UK; francisco.olmo@lshtm.ac.uk (F.O.); john.kelly@lshtm.ac.uk (J.M.K.); 3 LAPEN—Laboratory of Design and Synthesis of Chemotherapeutic Agents Potentially Active on Neglected Diseases, Department of Pharmacy, School of Pharmaceutical Sciences, University of Sao Paulo (USP), Sao Paulo 05508-9000, Brazil; elizabeth.igne@gmail.com; 4 Advanced Research Center in Medicine, School of Medicine, Union of the Colleges of the Great Lakes (UNILAGO), São José do Rio Preto, Sao Paulo 15030-070, Brazil

**Keywords:** Chagas disease, *Trypanosoma cruzi*, bioluminescence imaging, hydroxymethylnitrofurazone, benznidazole, trypanostatic effect

## Abstract

Hydroxymethylnitrofurazone (NFOH) is a therapeutic candidate for Chagas disease (CD). It has negligible hepatotoxicity in a murine model compared to the front-line drug benznidazole (BZN). Here, using *Trypanosoma cruzi* strains that express bioluminescent and/or fluorescent reporter proteins, we further investigated the in vitro and in vivo activity of NFOH to define whether the compound is trypanocidal or trypanostatic. The in vitro activity was assessed by exploiting the fluorescent reporter strain using wash-out assays and real-time microscopy. For animal experimentation, BALB/c mice were inoculated with the bioluminescent reporter strain and assessed by highly sensitive in vivo and ex vivo imaging. Cyclophosphamide treatment was used to promote parasite relapse in the chronic stage of infection. Our data show that NFOH acts by a trypanostatic mechanism, and that it is more active than BZN in vitro against the infectious trypomastigote form of *Trypanosoma cruzi*. We also found that it is more effective at curing experimental infections in the chronic stage, compared with the acute stage, a feature that it shares with BZN. Therefore, given its reduced toxicity, enhanced anti-trypomastigote activity, and curative properties, NFOH can be considered as a potential therapeutic option for Chagas disease, perhaps in combination with other trypanocidal agents.

## 1. Introduction

Chagas disease (CD) or American trypanosomiasis results from infection with the parasite *Trypanosoma cruzi* (*T. cruzi*). It is transmitted predominantly by different species of blood-sucking triatomine bugs [1,2]. Infections can also result from contaminated food or drink, blood transfusion, organ transplantation, and the congenital route [3,4]. Transmission of this parasitic zoonosis is restricted largely to Latin America, and is endemic in 21 countries, with approximately six million people infected [5,6]. Due to globalization, there has been an increase of cases in non-endemic areas, including the United States, Japan, Australia and Europe [7,8].

CD has a complex progression, divided into two distinct phases: acute and chronic. The acute phase, which can last between 2–8 weeks post-infection, is generally mild or asymptomatic. Parasites can be observed in the bloodstream and are widely distributed in organs and tissues. In some cases, symptoms such as fever and myalgia can appear, as well as myocarditis [9,10]. Once the acute phase is controlled by the immune system, the infection transitions to the life-long chronic stage [11,12]. Approximately 70% of those infected remain asymptomatic, however 20–30% develop symptomatic pathology, often decades later. This is characterized by irreversible heart damage and/or by digestive megasyndromes (megaesophagus or megacolon) [13,14].

CD treatment is limited to two nitroheterocyclic drugs, nifurtimox and benznidazole (BZN), both of which have been in use for more than 50 years. These drugs are effective against *T. cruzi* [15] during the acute stage of infection [16]. However, there is a limited cure rate during the chronic stage in both adults and children [17,18,19,20]. Currently, there are no new anti-chagasic compounds nearing approval for use in the clinic, and both BZN and nifurtimox can have serious side effects. Moreover, BZN can induce genome-wide mutagenesis and multi-drug resistance in *T. cruzi* [21]. Thus, limited efficacy and toxicity represent major challenges to be overcome if new treatments for CD are to be developed against this neglected disease, which remains a major public health issue in many areas of Latin America [22,23].

Hydroxymethylnitrofurazone (NFOH—a nitrofurazone derivative) is non-mutagenic in the Ames test [24], non-genotoxic in micronuclei [25], and pharmacokinetic studies in rats [26] and rabbits [27] have shown an increased distribution volume compared to its parent compound nitrofurazone. Additionally, when NFOH toxicity was evaluated after 21 and 60 days treatment, there was reduced hepatic inflammation compared to BZN, and no changes in hepatic enzymes such as aspartate aminotransferase and alanine aminotransferase [28,29]. Here, we report a comprehensive assessment of NFOH activity against *T. cruzi*. For in vitro analysis, we used the bioluminescent:fluorescent *T. cruzi* CL-Brener (TcVI) reporter strain [30], which allows for exquisite resolution in drug screening assays. For in vivo studies, we used the BALB/c murine model and bioluminescence imaging to monitor drug efficacy in the chronic stage of infection at high sensitivity. Our studies show that NFOH acts by a trypanostatic mechanism, shows significant activity against the infectious trypomastigote form than BZN, and can cure chronic infections.

## 2. Results

### 2.1. NFOH Has Greater Anti-Trypomastigote Activity Than BZN In Vitro

**Figure 1:**
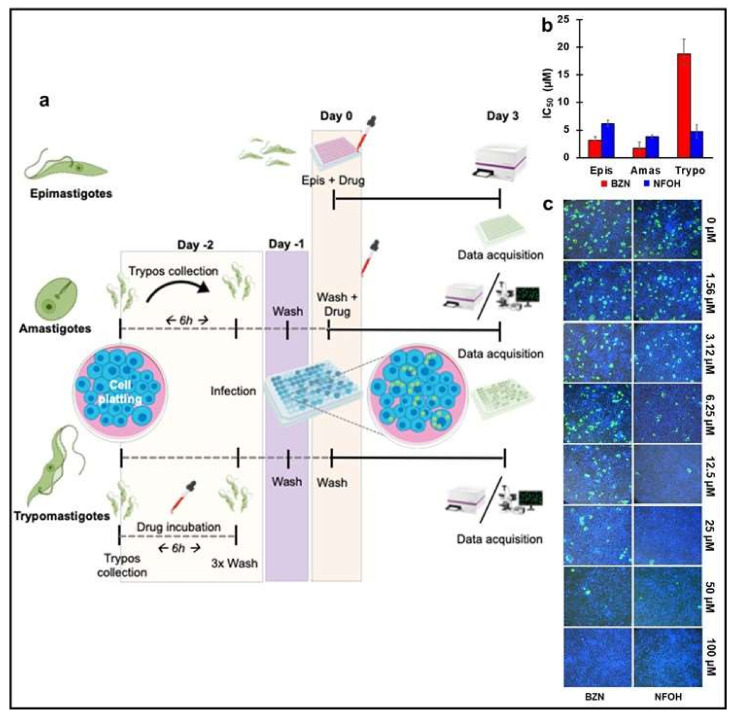
In vitro screening assays using bioluminescent:fluorescent *T. cruzi* (**a**) Schematic view of the screening protocols for measuring drug activity against epimastigote, amastigote and trypomastigote forms using *T. cruzi* CL-Luc::Neon [30]. (**b**) Drug activity against the three developmental forms of *T. cruzi*. The y axis indicates the IC_50_ values (µM) (see Materials and Methods for assay conditions). Values represent 3 individual replicates, and are the mean ± SD. (**c**) Image analysis of broad-field (4× captures from live fluorescent microscopy at the end-point of the trypomastigote screening assay, showing representatives of 30 separate images per treatment. Blue = Hoechst stain (1:10,000 added 2 h before imaging), Green = mNeonGreen fluorescent parasites.

To define NFOH anti-*T. cruzi* activity more clearly, primary whole-cell phenotypic screens of all three *T. cruzi* developmental stages were performed (Materials and Methods, Figure 1a). The data are based on eight-point potency curves, with BZN as the reference drug. Against replicative extracellular epimastigotes, BZN displayed slightly higher activity than NFOH, with an IC_50_ value of 3.2 µM compared to 6.1 µM (Figure 1b). With the more clinically relevant replicative intracellular amastigotes, the IC_50_ values were calculated using 3 days of exposure. This revealed a similar susceptibility profile, with values of 3.7 and 1.7 µM for NFOH and BZN, respectively (Figure 1b). Therefore, despite the drugs having to cross the host-cell membrane, both displayed significant activity with lower IC_50_ values under the assay conditions used.

To test against the invasive trypomastigote forms, which are nonreplicative, the IC_50_ was defined as the reduction of infection after drug exposure, rather than the inhibition of growth. This form of the parasite is responsible for the propagation of the disease within the mammalian host and also for its transmission to the insect vector during a blood meal. As can be inferred from the IC_50_ profile (Figure 1b), NFOH was four-fold more effective against these extracellular non-replicative forms than BZN. This significant decrease in infectivity could be also observed when the wells were imaged using real-time fluorescent microscopy (Figure 1c).

### 2.2. In Vitro Wash Out Assays Reveal the Trypanostatic Effect of NFOH

**Figure 2:**
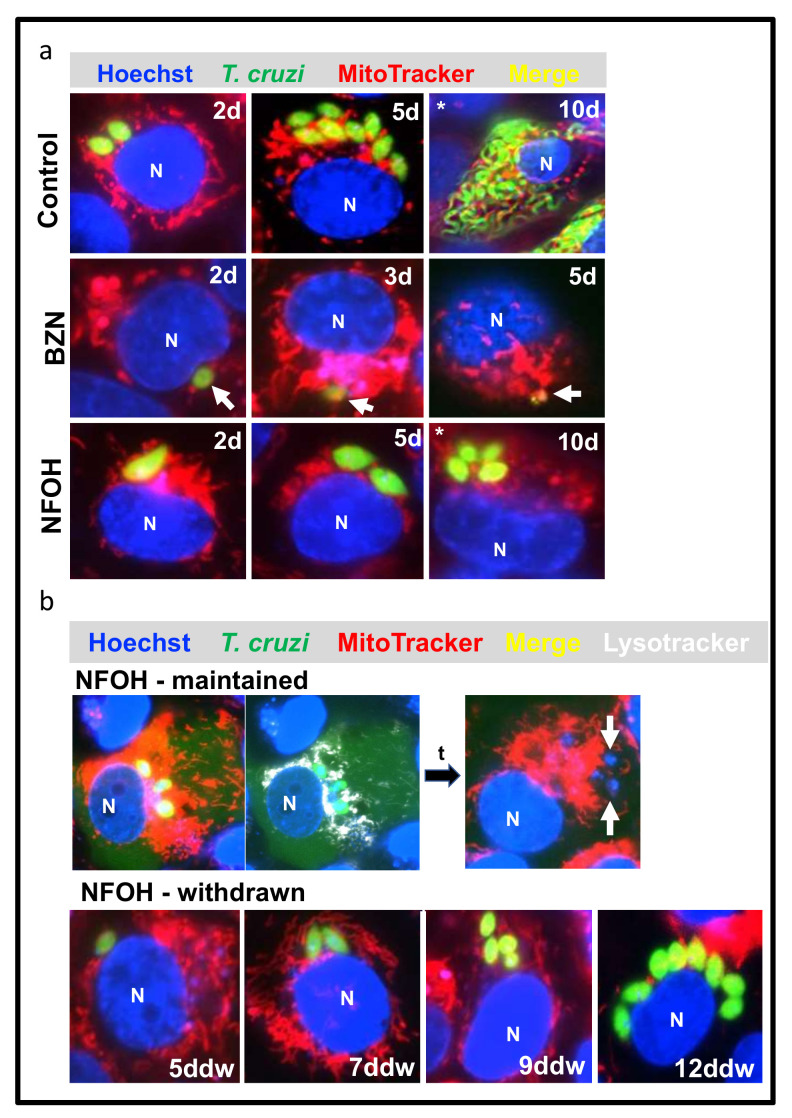
Infections of COLO-680N cells with *T. cruzi* CL-Luc::Neon were monitored by real-time fluorescent microscopy. (**a**) Fate of intracellular amastigotes after NFOH and BZN treatment. Cell monolayers were infected with trypomastigotes (Materials and Methods), and drug treatment was initiated 48 h later. Treatment with 10× IC_50_ BZN caused parasite death by day 3 post-treatment, with a strong reduction in fluorescence, followed by parasite disintegration by day 5 (white arrow). 10× IC_50_ NFOH treatment slowed replication, but did not lead to parasites destruction. Untreated parasites underwent replication and released differentiated trypomastigotes by day 10 (control). (**b**) Wash out assays. Cultures were treated for 24 days with 20× IC_50_ of NFOH, leading to blocked parasite replication (upper panel). Intense lysosomal activity was observed around parasites following labelling with lysotracker (Materials and Methods). This was associated with leakage of parasite green fluorescent protein into the host cell cytoplasm. When the NFOH treatment was stopped after 12 days, intracellular parasites re-initiated replication and completed several rounds by 12 days after NFOH withdrawal (ddw) (lower panel). N = mammalian cell nuclei. The images are representative of 20 individual infected cells monitored in real-time.

BZN requires dose- and time-dependent drug exposure to ensure that infections are completely resolved [31]. To further evaluate the potency of both drugs against replicative forms, we monitored the activity of BZN and NFOH by real-time fluorescent microscopy in vitro at a cellular level. We used 10 times the amastigote IC_50_ of each drug and followed cultures until untreated control infections began to release trypomastigotes (10–12 days) (Figure 2a). BZN caused parasite replication to cease by day 3 post-treatment initiation, and resulted in amastigote rupture by day 5. There was no parasite outgrowth, even when the drug was removed after the fifth day (Figure 2a). With 5 days NFOH treatment, a major reduction was observed in the replication rate, but once released from drug pressure, the proliferation re-initiated, and within the following 5 days, matched the levels in non-treated controls. To assess this further, parasites were treated with 20 times the IC_50_ of NFOH for 12 days, with the drug and medium renewed every 4 days. Following this, we observed that parasite replication had resumed within 5 days of drug withdrawal (Figure 2b), consistent with a trypanostatic mechanism. However, when treatment was maintained for an additional 12 days, this resulted in total parasite death. Lysotracker staining revealed high lysosomal activity in infected cells in the vicinity of remaining parasites, and leakage of green fluorescent protein into the cytoplasm of the host cell (Figure 2b).

### 2.3. NFOH Is More Effective against Chronic Stage T. cruzi Infections

**Figure 3:**
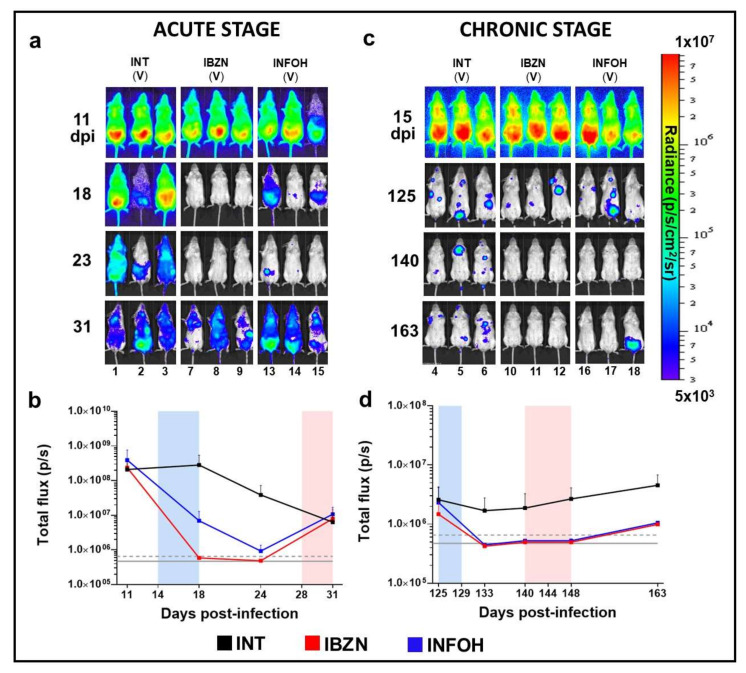
In vivo assessment of NFOH against acute (**a**,**b**) and chronic (**c**,**d**) stage *T. cruzi* infections. BALB/c mice were treated with NFOH or BZN at 100 mg kg^−^^1^ for 5 days (Materials and Methods) starting at 14 (acute) or 125 (chronic) days post-infection (dpi). (**a**,**c**) Representative ventral images of BALB/c mice acquired at various points dpi, as indicated. INT, infected non-treated (vehicle only); IBZN, infected treated with BZN; INFOH, infected treated with NFOH (*n* = 6 for acute and *n* = 12 for chronic stage infections). (**b**,**d**) Quantification of whole animal bioluminescence during the acute (**b**) and chronic (**d**) phase of infection (mean bioluminescence ± SEM) from all treated or non-treated mice. Grey horizontal solid line indicates mean background bioluminescence; dashed horizontal grey line indicates +2 SD (naïve mice, *n* = 4). Heat-maps are on log_10_ scales and indicate intensity of bioluminescence from low (blue) to high (red). Blue bar indicates five-day treatment period (**b**,**d**). All BZN- and NFOH-treated mice were immunosuppressed using cyclophosphamide during the period indicated by the pink bar (**b**,**d**) (Material and Methods).

**Figure 4:**
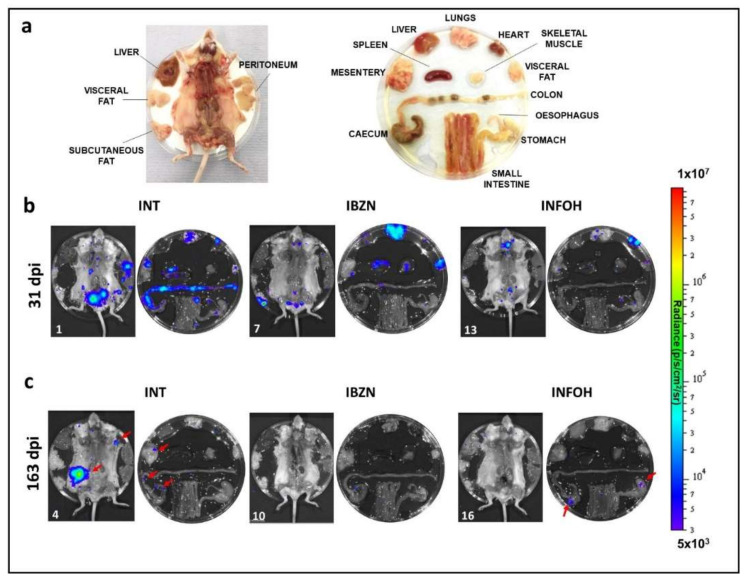
Ex vivo assessment of NFOH against acute and chronic stage *T. cruzi* infections. (**a**) Schematic display of carcass, organs and tissues. (**b**) Organs from representative mice treated during the acute stage were removed and assessed by bioluminescence imaging 31 days post-infection (dpi). INT, infected non-treated (vehicle only);IBZN, infected treated with BZN; INFOH, infected treated with NFOH. All mice were bioluminescence positive. See also Figure 3a. (**c**) Organs from mice removed 163 dpi following chronic stage treatment and immunosuppression (Figure 3c,d). Note the infection foci (red arrows) in the subcutaneous fat, skin, cecum, colon and mesentery in the non-treated mouse. The BZN-treated mouse was bioluminescence-negative and designated cured. The NFOH-treated mouse displayed infection foci (red arrows) in the stomach and cecum. The other three NFOH-treated mice examined were bioluminescence-negative, and designated cured.

Mice in the acute stage of infection were treated daily for 5 days (14 to 18 days post-infection) (dpi) via the oral route with BZN or NFOH (both at 100 mg kg^−^^1^). BZN treatment resulted in 99.8% knockdown in the parasite burden by day 18 (Figure 3a,b). This persisted until day 24. In parallel, NFOH treatment produced 97.5% knockdown by day 18, and by day 24, bioluminescence levels were close to background levels. At this point, the treated mice were immunosuppressed using cyclophosphamide (200 mg kg^−^^1^) (Materials and Methods). This led to recrudescence in all cases, as assessed by both in vivo (Figure 3a,b) and ex vivo imaging (Figure 4a,b).

NFOH was then tested against chronic stage infections, when the parasite load is much lower [32]. Mice were treated for five consecutive days with 100 mg kg^−^^1^, starting 125 dpi. This dosing regimen had been previously shown to produce a 100% cure rate with BZN [33,34], and the results achieved here were in line with this. The level of bioluminescence in mice treated with NFOH was reduced to background levels by 133 dpi (Figure 3c, d). Following cyclophosphamide-mediated immunosuppression, initiated on 140 dpi, in vivo imaging revealed that two of the NFOH-treated mice had relapsed after the last dose (Figure 3c,d). An additional mouse was found to be infected when examined by ex vivo imaging (Figure 4a,c). Therefore, 50% (3/6) of chronically infected mice treated with NFOH were assessed as cured.

## 3. Discussion

Nitroheterocyclic pro-drugs are subject to reductive metabolism in trypanosomatids, leading to the generation of reactive intermediates that are cytotoxic. The first step in this process is mediated by a type I nitroreductase (TcNTR-1) that catalyses a two-electron reduction [35]. Loss of TcNTR-1 activity results in increased resistance to a range of nitroaromatic drugs, including BZN and nitrofurazone. With BZN, drug metabolism leads to DNA damage and genome-wide mutagenesis [21,36,37,38,39,40]. Interestingly, NFOH is four times less mutagenic than its parent drug nitrofurazone [24], and does not induce genotoxic effects in micronucleus assays [25]. In the present work, we demonstrated that NFOH activity against *T. cruzi* in vitro had a trypanostatic effect over a short exposure time (Figure 2a,b). In the washout assay, activity depended more on the compound exposure time than the concentration used (Figure 2a,b). Importantly, we also revealed that it was superior to BZN in reducing trypomastigote infectivity (Figure 1b,c).

To better assess how these data translated into in vivo activity, we used a highly sensitive bioluminescence imaging model to monitor efficacy against acute and chronic infections. In the acute stage, although NFOH was non-curative under the conditions used (5 days dosing, 100 mg kg^−^^1^), it did reduce the parasite burden by >99%, similar to BZN, although this level of reduction took longer to achieve (Figure 3a,b). With chronic stage infections, there was a 50% cure rate (Figure 3c,d). In non-cured mice, NFOH treatment reduced the parasite load below detectable levels for more than 15 days, with immunosuppression required to promote parasite recrudescence. The greater curative activity against chronic infections could simply reflect the reduced parasite burden (10^2^–10^3^ fold) at this stage of the disease, with the trypanostatic effect of NFOH acting in concert with the immune response to eliminate the infection. Such synergism has been observed with BZN [41].

Combination therapy is now being considered as a viable strategy to control CD, given the toxicity of the front-line drugs, the current lack of curative options, and the possibility that “persister” parasites might act to limit drug efficacy. Combining NFOH and BZN, may at first glance appear counter-intuitive because the reductive activation of both agents is mediated by TcNTR1 [35]. However, NFOH produces fewer side effects, is less genotoxic, inhibits parasite replication by a different downstream mechanism, and as shown here, is considerably more active against the infectious trypomastigote form of the parasite. The reduced toxicity of NFOH, curative activity and potential for flexibility in terms of dosing regimens, also identifies it as an attractive partner for other anti-chagasic candidates in combination therapy [15,24,25,27,28,29,40,42].

## 4. Materials and Methods

### 4.1. Parasites and Cells Culture

*T. cruzi* CL-Luc::Neon epimastigotes were cultured in supplemented RPMI-1640 as described previously [30], and routinely maintained on selective agents (hygromycin, 150 μg mL^−^^1^ and G418, 100 μg mL^−^^1^). COLO-680N (human esophagus squamous cell carcinoma) cells (RRID:CVCL_1131) were cultivated to 95–100% confluency in Minimum Essential Medium Eagle (MEM, Sigma-Aldrich, Germany), supplemented with 5% Foetal Bovine Serum (FBS), 100 U mL^−^^1^ of penicillin, and 100 μg mL^−^^1^ streptomycin at 37 °C and 5% CO_2_. Tissue culture trypomastigotes (TCTs) were derived from previously infected COLO-680N cells. Cell cultures were infected for 18 h. External parasites were then removed by washing in Hank’s Balanced Salt Solution (Sigma-Aldrich, Germany), and the flasks incubated with fresh medium for a further 5–7 days. Extracellular TCTs were isolated by centrifugation at 1600 g. Pellets were re-suspended in Dulbecco’s Modified Eagle’s Medium (DMEM, Sigma-Aldrich, Germany) with 10% of FBS and kept at 37 °C until use. Motile trypomastigotes were counted using a haemocytometer.

### 4.2. In Vitro Screening

BZN and NFOH stocks were prepared in 100% dimethyl sulfoxide (DMSO) at 100 mM. Eight-point potency curves were generated with technical triplicates per assay in 96-well black polystyrene microplates. All potency determinations were performed as three independent experiments. Screening infections were done using a multiplicity of infection (MOI) of 10:1 (host cell:parasite). Trypomastigotes were isolated from a previous infective culture flask, incubated with different drug concentrations for 6 h, the drug was washed out and trypomastigotes were used for infection. Non-internalized parasites were removed from the medium on the following day and the infection monitored for an additional 96 h. Infectivity of treated trypomastigotes was measured using subsequent amastigote replication. Plates were incubated at 28 °C for epimastigotes assays, and 37 °C for the others with a 5% CO_2_ atmosphere. Fluorescence intensities were determined using a BMG FLUO star Omega (excitation 488 nm, emission 525 nm). Data were analysed using GraphPad Prism 8 software. Values are expressed as EC_50_ ± SD and are the average of three independent replicates.

### 4.3. Wash Out Assay Using Real Time Microscopy

In vitro infections for microscopy were carried out as above, but using eight-well Ibidi µ-slides with a polymer coverslip (Cat. No: 80826). Videos were acquired using an inverted Nikon Eclipse microscope. The slide containing the infected cells was moved along the x–y plane through 580 nm LED illumination. Images and videos were collected using a 16-bit, 1-megapixel Pike AVT (F-100B) CCD camera set in the detector plane. An Olympus LMPlanFLN 40 × 1.20 objective was used to collect the exit wave. Time-lapse imaging was performed by placing the chamber slide on a microscope surrounded by an environmental chamber (OKOLab cage incubator, Ambridge, PA, USA) maintaining the cells and the microscope at 37 °C and 5% CO_2_. Image sequences were created using the deconvolution app in Nikon imaging software.

### 4.4. Ethics Statement

Animal work was approved by the London School of Hygiene and Tropical Medicine Ethics Committee, under the UK Home Office project license (PPL 70/8207) in accordance with the UK Animals (Scientific Procedures) Act.

### 4.5. In Vivo Infection

*T. cruzi* CL Brener (TcVI) constitutively expressing the red-shifted firefly luciferase PpyRE9h were used in all infection experiments. Infectious metacyclic trypomastigotes and bloodstream trypomastigotes (BTs) were generated as previously described [32]. In standard experiments, 10^4^ in vitro-derived tissue-culture trypomastigotes or thawed cryopreserved BTs in 0.2 mL of phosphate-buffered saline (PBS) were first used to infect SCID mice via intraperitoneal (i.p.) inoculation. Parasitemic blood was obtained 2–3 weeks later and adjusted to 5 × 10^3^ BTs/mL with PBS. Female BALB/c mice aged 7–8 weeks were then infected with 10^3^ BTs via i.p. injection [32]. BALB/c mice were purchased from Charles River (UK) and maintained under specific pathogen-free conditions in individually ventilated cages, with a 12-h light/dark cycle and ad libitum food and water.

### 4.6. Treatment Schedule

BZN was synthesized by Epichem Pty Ltd., Australia, and NFOH (5-nitro-2-furaldehyde-N-(hydroxymethyl)-semicarbazone) was synthesized as described [42]. Both compounds, were prepared at 10 mg mL^−1^ in an aqueous suspension vehicle containing 5% (*v/v*) DMSO, 0.5% (*w/v*) hydroxypropyl methylcellulose, 0.5% (*v/v*) benzyl alcohol and 0.4% (*v/v*) Tween 80. BALB/c mice were treated with BZN or NFOH (100 mg kg^−^^1^) by oral gavage for 5 consecutive days. To detect parasite persistence, mice were immunosuppressed with 2 and 3 injections of cyclophosphamide (200 mg kg^−1^) by i.p. injection at 4-day intervals during acute and chronic phase of infection, respectively [33,34].

### 4.7. Bioluminescence Imaging

For in vivo bioluminescence imaging analysis, BALB/c mice were injected with 150 mg kg^−^^1^ D-luciferin i.p., then anaesthetized using 2.5% (*v/v*) gaseous isoflurane [33,34]. Five to ten minutes after d-luciferin administration, they were placed in an IVIS Lumina II system (Caliper Life Science, Waltham, MA, USA) and images acquired using LivingImage 4.3. Exposure times varied between 30 s and 5 min, depending on signal intensity. After imaging, mice were revived and returned to cages. For ex vivo imaging, mice were injected with D-luciferin and sacrificed by exsanguination under terminal anesthesia 5 min later. They were then perfused via the heart with 10 mL of 0.3 mg mL^−^^1^ D-luciferin in PBS. Organs and tissues were removed and transferred to a petri dish in a standardized arrangement, soaked in 0.3 mg mL^−^^1^ D-luciferin in PBS, and imaged using the maximum detection settings (5 min exposure for ventral and dorsal images, large binning). The remaining animal parts and carcasses were checked for residual bioluminescent foci, also using the maximum detection settings. The detection threshold for in vivo imaging was determined using control, uninfected mice [32]. Mice were designated cured if they were bioluminescence negative by both in vivo and ex vivo analysis following cyclophosphamide treatment.

### 4.8. Statistics

Statistical differences between groups were evaluated by using the Student’s *t*-test or one-way ANOVA, with Tukey’s post-hoc correction in GraphPad Prism v.7. Differences of *p* < 0.05 were considered significant.

## 5. Conclusions

In summary, NFOH is significantly more effective against infectious trypomastigotes than the front-line drug BZN. Against intracellular amastigotes, it acts by a trypanostatic mechanism. It is highly effective at reducing the parasite burden during acute stage *T. cruzi* infections (>99%) and can cure chronic infections, even with short-term-treatment (5 days).

## Data Availability

Not applicable.
